# Extended Finite Element Method with Simplified Spherical Harmonics Approximation for the Forward Model of Optical Molecular Imaging

**DOI:** 10.1155/2012/394374

**Published:** 2012-11-24

**Authors:** Wei Li, Huangjian Yi, Qitan Zhang, Duofang Chen, Jimin Liang

**Affiliations:** Life Sciences Research Center, School of Life Sciences and Technology, Xidian University, Xi'an, Shaanxi 710071, China

## Abstract

An extended finite element method (XFEM) for the forward model of 3D optical molecular imaging is developed with simplified spherical harmonics approximation (SP_*N*_). In XFEM scheme of SP_*N*_ equations, the signed distance function is employed to accurately represent the internal tissue boundary, and then it is used to construct the enriched basis function of the finite element scheme. Therefore, the finite element calculation can be carried out without the time-consuming internal boundary mesh generation. Moreover, the required overly fine mesh conforming to the complex tissue boundary which leads to excess time cost can be avoided. XFEM conveniences its application to tissues with complex internal structure and improves the computational efficiency. Phantom and digital mouse experiments were carried out to validate the efficiency of the proposed method. Compared with standard finite element method and classical Monte Carlo (MC) method, the validation results show the merits and potential of the XFEM for optical imaging.

## 1. Introduction

Light propagation model in biological tissue is the foundation of optical imaging. An accurate forward model is important for location and quantification of target distribution in the fields of optical imaging modalities, such as diffusion optical tomography (DOT), fluorescence molecular tomography (FMT), bioluminescence tomography (BLT), and Cerenkov luminescence tomography (CLT) [1–5]. The propagation of the emission photons in tissue can be accurately represented by the radiative transfer equation (RTE) or Monte Carlo (MC) models, but they are extremely computationally expensive. Therefore, the commonly used mathematical model in optical imaging field is the diffusion approximation (DA) to RTE. However, the DA model can be used only in the highly scattering property of the biological tissue, and is not suitable for the real mouse with complex internal tissues. To reach a compromise between the accuracy and efficiency, simplified spherical harmonics approximation (SP_*N*_) to RTE is employed due to its capacity in improving the solution in transport-like domains with high absorption and small geometries [6, 7]. 

Owing to the complex and curvilinear geometries associated with the biological tissues, the classical finite element methods (FEM) with SP_*N*_ approximation become necessary for optical imaging, especially for heterogeneous tissues [8–10]. In the FEM scheme, the region of heterogeneous tissue is divided into small tetrahedron elements. The linear functions of the tetrahedron element are employed in the standard finite element basis function which requires the homogeneity of tissue in one element. The fine triangle mesh between the two tissues is required to conform to the complex internal boundary for ensuring the calculation accuracy of FEM. However, the generation of the internal boundary mesh is a hard work and time consuming task. Moreover, the fine mesh yields the excess cost in finite element computation, especially with SP_*N*_ approximation in three dimensions. Fortunately, the extended finite element method inherits all the advantages of standard FEM and exempts the internal boundary mesh generation. Consequently, the extend-finite element method (XFEM) may deal with the problems of fine triangle mesh generation perfectly. 

XFEM was first introduced in the literature [11], and it has many applications in the area of mechanics [11, 12]. To the best of our knowledge, it has not been used with SP_*N*_ approximation for optical imaging. In this study, we establish the mathematical framework of XFEM with SP_*N*_ approximation as the optical forward model. Specifically, a mesh without the internal boundary conformation is employed in the XFEM scheme. The distance relationship between the tetrahedron vertex and the real tissue boundary is reflected by a signed distance function which is used to construct the enriched basis function. Then the enriched function is added to the standard finite element basis function, therefore a standard approximation of FEM is thus “enriched” in the discretized region (usually cut by the internal tissue boundary) of interest. In the weak form of SP_*N*_ equations, Gaussian quadrature is employed to calculate the integrals and the linear system equations are established. As a result, the calculation of XFEM can be carried out without the time-consuming internal boundary mesh generation. Moreover, the required overly fine mesh conforming to the complex tissue boundary which leads to excess time cost can be avoided. XFEM conveniences its application to tissues with complex internal structure and improves the computational efficiency. Numerical experiments with a phantom and a digital heterogeneous mouse were carried out to evaluate the performance of the proposed method. The results were compared with that of standard FEM and MC method to demonstrate the efficiency of XFEM. 

The paper is organized as follows. In Section 2, the detailed procedure of using XFEM for solving SP_*N*_ equations is derived. In Section 3, we evaluate the performance of the proposed method by comparing with the standard FEM and MC method in the experiments, and demonstrate the efficiency of the method. Conclusions and discussions are given in the last section.

## 2. Method

### 2.1. SP_*N*_ Approximations

The general form of the (*N* + 1)/2  SP_*N*_ equations and its (*N* + 1)/2 boundary conditions for optical imaging in three dimensions are [13]:

(1) −∇·Ci,∇φi(r)∇φi(r)+∑j=1(N+1)/2Ci,φi(r)φi(r)  =Ci,Q(r)Q(r), r∈Ω,  i,j∈[1,(N+1)2].∑j=1(N+1)/2Ci,∇φjb(r)n⃑·∇φj(r)  =∑j=1(N+1)/2Ci,φjb(r)φj(r)+Ci,SSi(r),       r∈∂Ω,i,j∈[1,(N+1)2],

where *φ*_*i*_(*r*) is the SP_*N*_ composite moments of the radiances in RTE, and *C*_*i*,∇*φ*_*i*__(*r*), *C*_*i*,*φ*_*i*__(*r*), *C*_*i*,*Q*_(*r*), *C*_*i*,∇*φ*_*j*__^*b*^(*r*), *C*_*i*,*φ*_*j*__^*b*^(*r*), and *C*_*i*,*S*_ denote the coefficients which are related to ∇*φ*_*i*_(*r*), *φ*_*i*_(*r*), *Q*, and *S* for SP_*N*_ equations at each point *r* in the region *Ω* or boundary ∂*Ω*. These can be calculated by the absorption coefficient *μ*_*a*_[mm^−1^], scattering coefficient *μ*_*s*_[mm^−1^], anisotropy parameter *g*, and refractive index *n*. *Q*(*r*) is the internal source, and it represents the bioluminescence source in BLT or the fluorophore in and FMT. *S*_*i*_(*r*) is the external source which represent the external laser source in DOT and FMT. $\overset{⃑}{n}$ represents the unit normal vector outward to the boundary.

The exiting partial current *J* ^+^[nW/mm^2^] can be obtained from detector readings at the tissue boundary ∂*Ω* which can be acquired by CCD camera in practical application, and we have the general formulation:

(2) J+=∑j=1(N+1)/2C∇φjJ(r)n⃑·∇φj(r)+∑j=1(N+1)/2CφjJ(r)φj(r),

where *C*_∇*φ*_*j*__^*J*^(*r*) and *C*_*φ*_*j*__^*J*^(*r*) are the coefficients which can be calculated for SP_*N*_ equations. The detailed derivation of (1)–(2) refers to the literature [13].

### 2.2. Extended Finite Element Discretization

**Figure 1:**
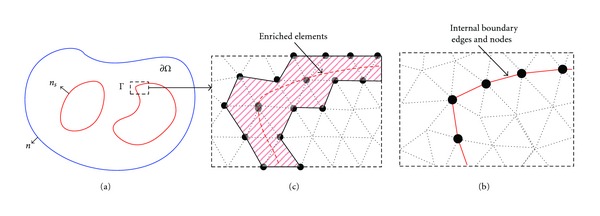
(a) Heterogeneous tissues. (b) Standard finite element mesh boundary conforms to the interface. (c) Enriched elements and nodes of XFEM “cut” by the interface.

The SP_*N*_ equations and its boundary conditions can be solved using Galerkin finite element scheme, the weak form of SP_*N*_ equations can be written as follows:

(3) ∫ΩCi,∇φi(r)·∇φi(r)·∇v(r)dr  +∑j=1(N+1)/2∫ΩCi,φj(r)φj(r)·v(r)dr  −∑j=1(N+1)/2∫∂ΩCi,∇fφj(r)∇fn·φi(φj(r))·v(r)dr =∫ΩCi,Q(r)Q(r)·v(r)dr   +∫∂ΩCi,fSi(r)fSi(Si(r))·v(r)dr,

where *f*_*n*·*φ*_*i*__(*φ*_*j*_(*r*)) and *C*_*i*,*f*_*S*_*i*___(*r*) are the coefficient matrices with respect to *φ*_*j*_(*r*) and *f*_*S*_*i*__(*S*_*i*_(*r*)). *v*(*r*) is the test function, and it is the same as the standard linear basis function in elements. For finite element analysis in three dimensions, tetrahedral elements have become popular in numerical computation, because of their ability to describe complex geometries such as heterogeneous tissues. Thus the volumetric domain is discretized into small tetrahedral elements, and the elements need to conform to the internal tissue boundary as shown in Figures 1(a) and 1(b). When the internal boundary is not smooth, the size of the element must be small enough to ensure the accuracy and this may cause difficulty in mesh generation and lead to huge computation burden. In XFEM framework, mesh is generated as a region consists many different tissues, and the elements “cut” by the actual tissue boundary are enriched as shown in Figures 1(a) and 1(c). Using the enriched basis functions constituted by the signed distance functions, the boundary can be determined.

### 2.3. Enriched Strategy

In XFEM scheme, the signed distance function is adopted to depict the internal boundary of different tissues. The definition of the signed distance function *N*(*r*) is

(4) N(r)=min⁡rb∈Γ||r−rb||sign⁡(ns·(r−rb)).sign⁡(ξ)={1  if  ξ≥0,−1 if  ξ<0,

where *r* ^*b*^ is the point at the internal boundary Γ, sign⁡ is the signed function, and *n*_*s*_ denotes the unit normal vector outward to the internal boundary as shown in Figure 1(a). Herein, the position relation between the discrete point and the continuous boundary is completely reflected by the function *N*(*r*).

The enriched basis function *ψ*_*j*_(*r*) composed of the signed distance function *N*(*r*) of the element at tetrahedron node *j* can be derived, and the continuous *N*(*r*) is discretized with its value at the node. The enrichment functions introduce a discontinuity in the gradient of the radiances field *φ*_*i*_(*r*) or the distribution of optical parameters to Γ, thus the following integral of enriched function is more accurate than that of linear basis function:

(5) ψj(r)=vj(r)(|N(r)|−|Nj|).N(r)=∑k=14vk(r)Nk.Nj=N(rj),

where *v*_*j*_(*r*) is the linear basis function of the tetrahedron element. Using the enriched basis function *ψ*_*j*_(*r*), the enriched approximation $\overset{-}{\varphi }(r)$ can be written as the following form:

(6) φ(r)≈∑i=1Ncϕivi(r).φ−(r)≈φ(r)+∑j=1Neajψj(r)≜v−(r)φ−,

where *φ*(*r*) is the conventional approximation and *N*_c_ is the number of nodes. $\overset{-}{\varphi }(r)$ is the extended approximation, *a*_*j*_ is the enriched degrees of freedom, and *N*_e_ is the number of the enriched nodes. It is clear that $\overset{-}{\varphi }(r)$ includes *φ*(*r*) as a special case, and each enriched element has eight degrees of freedom. Rewrite the extended linear basis functions and the discrete point value of $\overset{-}{\varphi }(r)$ in matrix form, we obtain

(7) v−(r)=[v−1(r),v−2(r)⋯v−Nc+Ne(r)]=[v1(r),v2(r)⋯vNc(r),ψ1(r),ψ2(r)⋯ψNe(r)].φ−i=[ϕi,1,ϕi,2⋯ϕi,Nc,ai,1,ai,2⋯ai,Ne]T,            i∈[1,(N+1)2],

where $\overset{-}{v}(r)$ and ${\overset{-}{\varphi }}_{i}$ have *N*_c_ + *N*_e_ components for each variable of SP_*N*_ equations. This form can directly use to assemble the finite element matrix.

### 2.4. Integrals and System Matrix

For finite element discretization, the matrix form of the (*N* + 1)/2SP_*N*_ equations (3) can be written as follows:

(8) ∑elem[m1,φ−1m1,φ−2⋯m1,φ−(N+1)/2m2,φ−1m2,φ−2⋯m2,φ−(N+1)/2⋮⋮⋮⋮m(N+1)/2,φ−1m(N+1)/2,φ−2⋯m(N+1)/2,φ−(N+1)/2] ×[φ−1φ−2⋮φ−(N+1)/2]=∑elem[b¯1b¯2⋮b¯(N+1)/2],

where elem denotes all the elements in the region, for the (*N* + 1)/2 variables $\overset{-}{\varphi }$ and $\overset{-}{b}$, each of which has *N*_c_ + *N*_e_ components in total according to (7). $m_{i,{\overset{-}{\varphi }}_{j}}$ in the left coefficient matrix is the small element matrix and can be written in details:

(9) mi,φ−j={∫ΩeCi,∇φi(r)·∇v−p·∇v−qdr+∫ΩeCi,φj(r)v−p·v−qdr−∫∂ΩCi,∇fφj(r)fn·φi(v−p)·v−qdr, i=j.∫ΩeCi,φj(r)v−p·v−qdr−∫∂ΩCi,∇fφj(r)fn⃑·φi(v−p)·v−qdr, i≠j,

where ${\overset{-}{v}}_{p}$ and ${\overset{-}{v}}_{q}$ are the corresponding matrix elements of $\overset{-}{v}(r)$ in (7) in the finite element *Ω*_e_. *p*, *q* are the number marks of the four points in element *Ω*_e_. Similarly, ${\overset{-}{b}}_{i}$ in the right is

(10) $$\begin{array}{c} {\overset{-}{b}}_{i}=\int_{\Omega }C_{i,Q}\left(r\right){\overset{-}{Q}}_{p}\cdot {\overset{-}{v}}_{q}dr+\int_{\partial \Omega }C_{i,f_{S_{i}}}\left(r\right)f_{S_{i}}\left(S_{i}\left(r\right)\right)\cdot {\overset{-}{v}}_{q}dr. \end{array}$$

**Figure 2:**
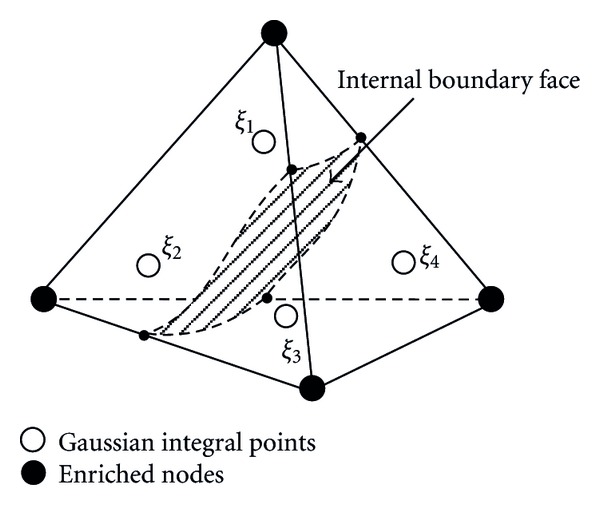
Gaussian quadrature in an enriched element.

In finite element framework, for ease of calculation, the integrand is assumed continuous, and the Gaussian quadrature can be used. In this paper, on the one hand, exact integrals can be obtained for the standard linear elements. On the other hand, the second order Gaussian quadrature with four quadrature points is adopted for the remaining enriched elements as showed in Figure 2. The Gaussian quadrature formula in the standard element *Ω*_e_ is

(11) $$\begin{array}{c} \int_{\Omega_{\text{e}}}F\left(r\right)dr\approx \sum_{i=1}^{4}G_{i}F\left(\xi_{i}\right), \end{array}$$

where *F*(*r*) is the general integrand, *G*_*i*_ is the coefficient and all is 0.25, *ξ*_*i*_ is the Gaussian quadrature point as showed in Figure 2.

For the integrand *F*(*r*), the integrals of enriched function can be calculated by the linear basis function *v*_*j*_(*r*), and *v*_*j*_(*r*) at the Gaussian points is a constant as follows:

(12) ∇ψj(r)|r=ξi  =∇vj(r)·(|N(r)|−|Nj|)   +vj(r)·∇(|N(r)|−|Nj|)|r=ξi.N(r)|r=ξi=∑k=14vk(ξi)Nk.vj(ξi)=0.5854, i=j.vj(ξi)=0.1382, i≠j.

Incorporating (7) and (8), and assembling all the element matrixes (8), after using the Gaussian quadrature to calculate the enriched elements of (9), the linear system equations of SP_*N*_ equations is established and can be rewritten in the matrix form:

(13) M(Nc+Ne)∗(N+1)/2,(Nc+Ne)∗(N+1)/2Φ−(Nc+Ne)∗(N+1)/2,1 =B−(Nc+Ne)∗(N+1)/2,1,

where *M* is a matrix including (*N*_c_ + *N*_e_)∗(*N* + 1)/2 rows and (*N*_c_ + *N*_e_)∗(*N* + 1)/2 columns, $\overset{-}{\Phi }$ is the unknowns including (*N*_c_ + *N*_e_)∗(*N* + 1)/2 components, and $\overset{-}{B}$ is the source term including (*N*_c_ + *N*_e_)∗(*N* + 1)/2 components. Obtain the $\overset{-}{\Phi }$ of (13) and instead of *φ*(*r*) in (2). The linear relationship between the surface detector readings *J* ^+^ and the source *S* or *Q* is established.

The goal of forward problem is to get the relationship between the surface detector readings and the internal sources. To have the general and particular comparison, the correlation coefficient CORR(*J*_*X*/*F*_^+^, *J*_Fine_^+^) and the well known mean relative numerical error MRNE_Fine_ of *J* ^+^ are both defined to quantitatively evaluate the performance of XFEM or FEM on the coarse mesh with respect to the FEM on the fine mesh:

(14) CORR(JX/F+,JFine+)=∑i=1Ns(Ji,X/F+−J−i,X/F+)(Ji,Fine+−J−i,Fine+)(∑i=1Ns(Ji,X/F+−J−i,X/F+)2)(∑i(Ji,Fine+−J−i,Fine+)2),

(15) $$\begin{array}{c} \text{MRN}{\text{E}}_{\text{Fine}}=\frac{\sum_{i=1}^{N_{\text{s}}}\left(J_{i,X/F}^{+}-J_{i,\text{Fine}}^{+}\right)/J_{i,\text{Fine}}^{+}}{N_{\text{s}}}, \end{array}$$

where *J*_*i*,*X*/*F*_^+^ is the *J* ^+^ compute by XFEM or FEM, *J*_*i*,Fine_^+^ is compute by FEM on the fine mesh. *N*_s_ is the number of the sampling point, ${\overset{-}{J}}_{i,X/F}^{+}$ and ${\overset{-}{J}}_{i,\text{Fine}}^{+}$ is mean value of *J*_*i*,*X*/*F*_^+^ *J*_*i*,Fine_^+^ in (14). CORR(*J*_*X*/*F*_^+^, *J*_Fine_^+^) = 1 illustrates the two data is identical after normalization and can be used to assess the degree of closeness between the two data. Similarly, by substituting the *J*_*i*,MC_^+^ for the *J*_*i*,Fine_^+^ in (14) and (15), the mean relative numerical error MRNE_MC_ and correlation coefficient CORR(*J*_*X*/*F*/MC_^+^, *J*_MC_^+^) of XFEM and FEM with respect to MC method can be obtained for evaluation.

## 3. Results and Discussion

### 3.1. Regular Phantom Experiment Compared with Standard FEM

**Figure 3:**
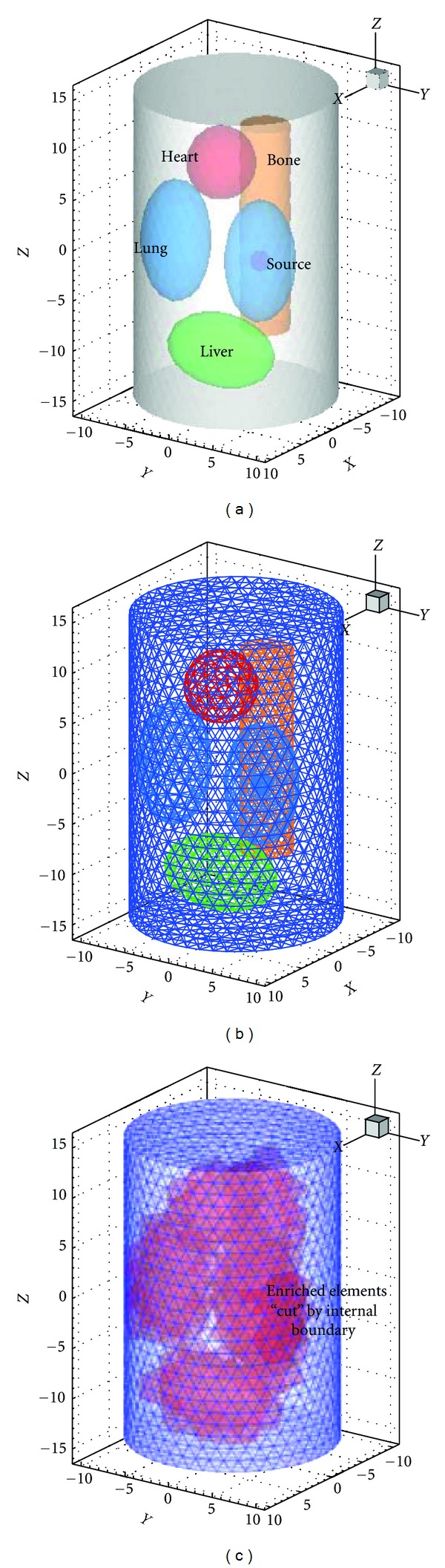
(a) Heterogeneous phantom, (b) the element mesh of FEM, and (c) element mesh of XFEM without internal boundary generation, red region is the enriched region.

The validation studies were performed using a cylindrical phantom of 30 mm height and 10 mm radius to model a mouse. It consisted of ellipsoids or cylinders to represent the tissues of mouse as shown in Figure 3(a). A solid sphere source of 1 mm radius and 0.238 nW/mm^3^ power density was centered at (3, 5, 0) inside the right lung. The relevant optical parameters from literature [14] are listed in Table 1. Numerical simulations are carried out to compare the standard FEM and XFEM for SP_*N*_ equations. The coarse mesh containing 3459 nodes without internal mesh boundary generation was used for XFEM and another coarse mesh contained 3573 nodes for FEM as shown in Figures 3(b) and 3(c). Because the precise analytic solutions for heterogamous phantom is difficult to obtained, the FEM on the fine mesh can get relative accurate solution according to the classical finite element analysis:

(16) $$\begin{array}{c} ||\varphi -\varphi^{h}||\to 0\;\text{as}\;\;h\to 0, \end{array}$$

where the numerical solution *φ* ^*h*^ of FEM converges to the exact solution *φ* as the mesh size *h* decreases. We choose the result of the FEM on the fine mesh containing 12312 nodes as the standard for comparison. The program of FEM and XFEM is coded in MATLAB on the desktop computer (Intel(R) Xeon(R) 2 CPU E5430 @ 2.66 GHz, and 8 G RAM).

**Figure 4:**
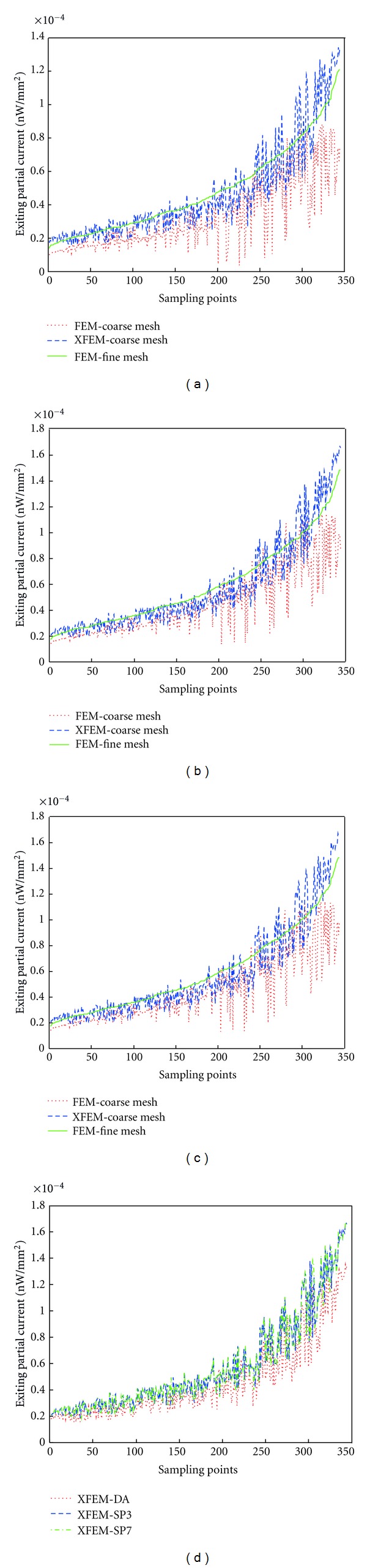
Surface detector readings *J* ^+^ for SP_*N*_ equations around the regular heterogeneous phantom using the two methods on the coarse mesh compared with the FEM on the fine mesh. (a) DA equations. (b) SP_3_ equations. (c) SP_7_ equations. (d) Comparison among the results of XFEM for DA, SP_3_, and SP_7_ equations.

345 interpolate points whose value is nonzero are uniformly sampled around the phantom surface. Choosing the FEM on the fine mesh as the standard, the absolute value of exiting partial current *J*_*X*/*F*_^+^ on the sampling points are arranged in ascending order. Then the results of XFEM, and FEM on the comparable coarse mesh at these sampling points can be obtained by interpolation. The comparison results with Diffusion, SP_3_, SP_7_ approximations are showed in Figures 4(a), 4(b), and 4(c), respectively. Although the curve has fluctuation caused by the discretization error, the results have similar tendency. It is clear that the blue curve of XFEM on coarse mesh is closer to the green curve of FEM on fine mesh than that of FEM on the coarse mesh. Compared with the FEM, the CORR(*J*_*X*/*F*_^+^, *J*_Fine_^+^) of XFEM is closer to 1 for DA, SP_3_, and SP_7_ with 0.94, 0.95, and 0.90. The two methods is carried out on comparative mesh even the XFEM on the slightly less coarse mesh (3459 nodes). Thus the solution using XFEM is nearer to the true solution. The MRNE_Fine_ of XFEM is 14%, 15%, and 16%, which is much smaller than FEM, whose MRNE_Fine_ is 31%, 22%, and 22% for DA, SP_3_, and SP_7_ equations respectively. The results listed in Table 2 using the two evaluation indexes demonstrate the validity of the proposed method for SP_*N*_ equations. Moreover, it indicates that the XFEM is superior to the standard FEM and the solution of XFEM is more closer to the true solution for SP_*N*_ equations.

### 3.2. Digital Mouse Experiment Compared with Monte Carlo Method

**Figure 5:**
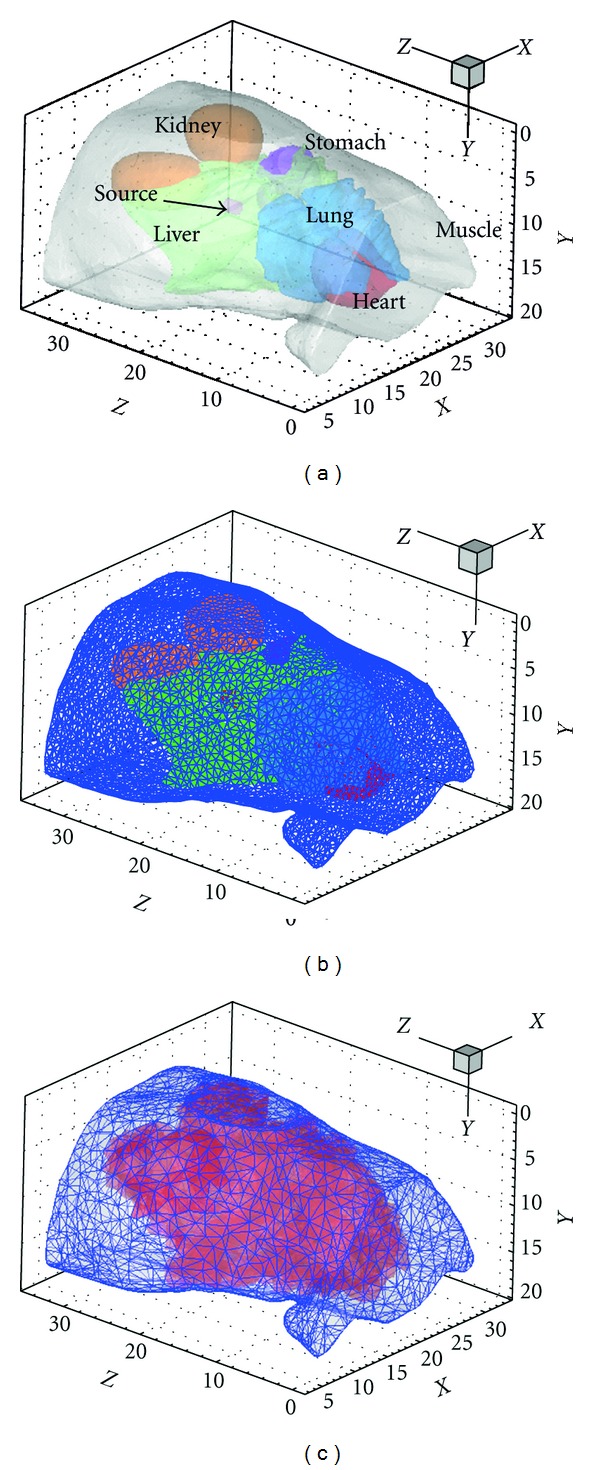
(a) The digital mouse model. (b) The external and internal boundary mesh for FEM. (c) The external boundary mesh and enriched region for XFEM.

In this experiment, a digital heterogeneous mouse from CT and cryosection data consisting of several organs is adopted to evaluate the performance of the XFEM. The mouse is shown in Figure 5(a) [15]. A cylinder source of 0.8 mm radius, 1.6 mm height, and 0.311 nW/mm^3^ power density was centered at (12, 7.5, 50.5) inside the liver. The optical parameters of the organs at 670 nm wavelength computed by the literature [14] are employed and shown in Table 3. The experiments were conducted using XFEM on the coarse mesh, FEM on the fine mesh, and classical MC method. The surface detector readings using MC method was achieved from MOSE [16] using 5 million photons. Since there are still errors between the SP_*N*_ equation and MC to depict the light propagation, the XFEM is compared with the FEM and MC simultaneously.

**Figure 6:**
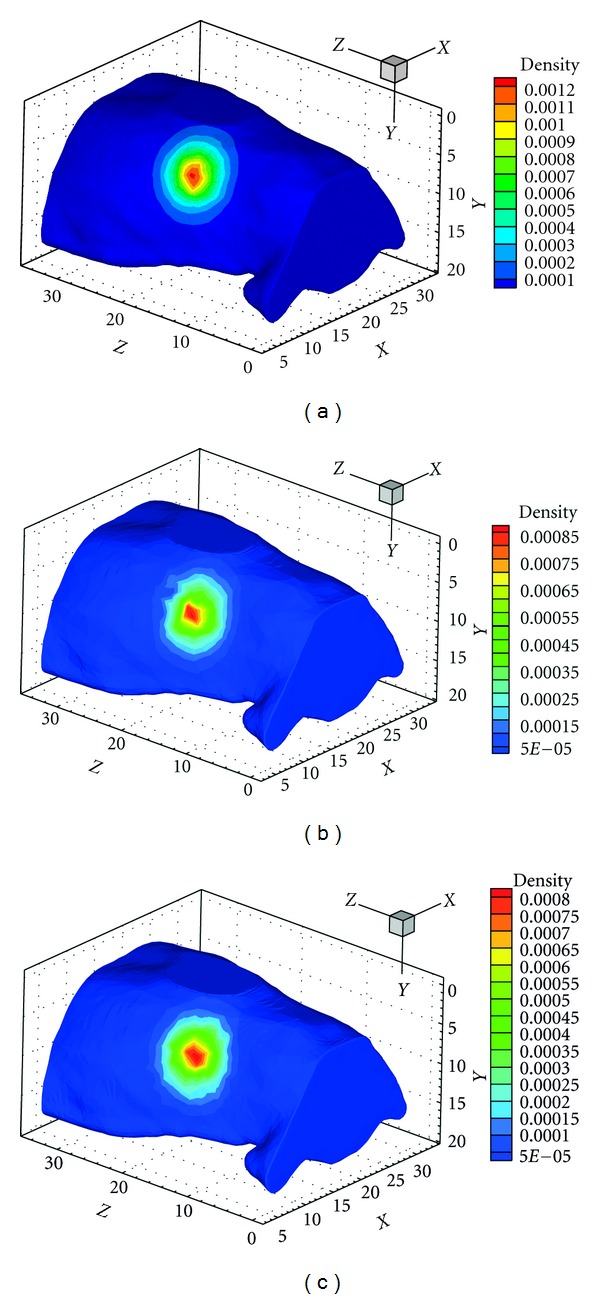
The surface detector readings with SP_3_ approximation (a) using FEM on the fine mesh, (b) using XFEM on the coarse mesh, and (c) using MC method.

**Figure 7:**
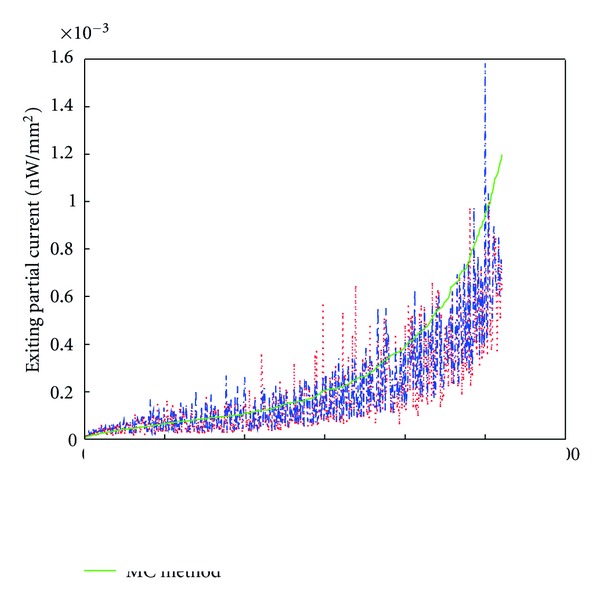
Surface detector readings *J* ^+^ using MC method, XFEM, and FEM at the sampling points. Green line denotes the results of MC method, red and blue lines are that of FEM and XFEM with SP_3_ approximation.

As shown in Figure 4(d), it is clear that the blue and green curves agree well. Considering the difference of the results between SP_3_ and SP_7_ equations are very small, for simplicity, only the SP_3_ results are presented in Figure 6. The absolute values on the surface using the FEM, XFEM and MC methods agree well and have the similar tendency in general. For detailed comparison, 521 points with nonzero values on the mouse surface are sampled, and then the surface value of these points using the three methods are obtained by interpolation and shown in Figure 7. Choosing the MC method as standard, the mean relative numerical error MRNE_MC_ and correlation coefficient CORR(*J*_*X*/*F*/MC_^+^, *J*_MC_^+^) of XFEM and FEM with respect to MC method are obtained and shown in Table 4. The MRNE_MC_ and CORR(*J*_*X*/*F*/MC_^+^, *J*_MC_^+^) of FEM is 0.86 and 44%, while those of XFEM is 0.93 and 45%. The computational time of several modules that perform specific computational tasks, mesh generation, matrix assembly and solver. Since the XFEM does not have the complex mesh generation as FEM, the total time cost of matrix assembly and solver is considered for comparison. It is clear that the time cost of XFEM is only 367 seconds on the coarser mesh while that of FEM is 2675 seconds with the similar accuracy results. All the time cost of two methods are far less than that of MC method. The XFEM has a distinct strength on time-efficiency and this makes it more practical in imaging process.

## 4. Conclusion

We have derived the extended finite element method with SP_*N*_ approximations for the forward model of the three dimensional optical imaging. Considering the complex geometric object, it is necessary to have the fined mesh to conform to the internal boundary. And the mesh conformation is a difficult issue in the pretreatment in the FEM, moreover the standard FEM on the fine mesh for SP_*N*_ approximations cost too much especially for the high order approximation. Fortunately, the XFEM can deal with this problem. The XFEM includes the standard FEM as a special case, which does not require a geometric representation of the interface or any boundary mesh generation. Use the signed distance functions add to the standard basis functions, the number of nodes can keep unchanged. The interface can be well depicted by the enriched functions, also the solution of SP_*N*_ equation is more accurate.

The XFEM is validated through the numerical experiments. Phantom experiment was conducted and its results agreed well with the well known classical FEM. Moreover, compared with the FEM, XFEM can get more accurate result even on the slightly coarse mesh, which is closer to the FEM on the fine mesh. Digital mouse experiments further indicate that XFEM is superior to the standard FEM. The MC method was employed to evaluate the performance the proposed method. Although the relative errors and correlation coefficient using the XFEM with respect to MC method is comparable to that of standard FEM, the time cost of XFEM is greatly decreased due to the adoption of coarser element mesh. All these indicate that the XFEM is more suitable for SP_*N*_ equations especially in complex heterogeneous tissues.

Adaptive FEM method is often adopted for its high efficiency [17, 18]. But nearly all the adaptive method is based on the mesh refinement which is extremely complicate especially in three dimensions. XFEM can also be seen as an adaptive FEM method because it increases the degrees of freedom near the internal boundary while using fixed mesh, therefore the mesh refinement can be avoided. Thus the process in each adaptive level is simplified and the calculation cost is decreased. Moreover, the discretization error of the forward model is improved, which is important for the quantification reconstruction. XFEM may provide a potential tool for reconstruction algorithm.

## Figures and Tables

**Table 1:** Optical parameters of phantom.

Material	*μ*_*a*_[mm^−1^]	*μ*_*s*_[mm^−1^]	*g*
Muscle	0.01	4.0	0.9
Lung	0.35	23.0	0.94
Heart	0.2	16.0	0.85
Liver	0.002	20.0	0.9
Bone	0.035	6.0	0.9

**Table 2:** Results of XFEM and FEM for SP_*N*_ approximations.

Method		FEM	XFEM
Number of nodes		3459	3573
Number of elements		15540	15987

	DA	0.85	0.94
CORR(*J*_*X*/*F*_^+^, *J*_Fine_^+^)	SP3	0.88	0.95
	SP7	0.88	0.90

	DA	31%	16%
MRNE_Fine_	SP3	22%	14%
	SP7	22%	15%

**Table 3:** Optical parameters of the mouse organs.

Organs	*μ*_*a*_[mm^−1^]	*μ*_*s*_[mm^−1^]	*g*
Muscle	0.08697	4.29071	0.90
Heart	0.05881	6.42581	0.85
Stomach	0.01139	17.96150	0.92
Liver	0.35182	6.28066	0.90
Kidney	0.06597	16.09293	0.86
Lung	0.19639	36.23141	0.94

**Table 4:** Comparison among the FEM, XFEM and MC method.

Method	FEM	XFEM	MC method
Number of nodes	24906	6541	/
Number of elements	132202	32398	/
CORR(*J*_*X*/*F*/MC_^+^, *J*_MC_^+^)	0.86	0.92	1
MRNE_MC_	44%	45%	0
Time cost [*s*]	2675	367	7292
